# The red hat — designating leadership using visual and verbal cues: a mixed-methods study

**DOI:** 10.1186/s41077-024-00295-2

**Published:** 2024-07-03

**Authors:** Kelli Krase, Julie A. Broski, Stephen Tarver, Shariska P. Harrington, Amy Wolverton, Mae Winchester, German Berbel, Melody K. Zakarian, Taylor Zabel, Hannah Warren, Matthew C. Lineberry

**Affiliations:** 1 Department of Obstetrics and Gynecology, The University of Kansas School of Medicine, Kansas City, KS USA; 2 The Zamierowski Institute for Experiential Learning, The University of Kansas Medical Center, Kansas City, KS USA; 3 Department of Anesthesiology, Pain and Perioperative Medicine, The Zamierowski Institute for Experiential Learning, The University of Kansas Medical Center, Kansas City, KS USA; 4 Department of Obstetrics and Gynecology, Minnesota Oncology, St. Paul, MN USA; 5 Department of Obstetrics and Gynecology, University Hospitals Cleveland Medical Center, Cleveland, OH USA; 6 Department of Surgery, The University of Kansas School of Medicine, Kansas City, KS USA

**Keywords:** Simulation, Communication, Obstetric emergencies, Interprofessional simulation, Critical event, Crisis resource management

## Abstract

**Background:**

During a critical event in the labor and delivery operating room, it is crucial for team members responding to the situation to be aware of the designated leaders. Visual and verbal cues have been utilized to designate leadership in various healthcare settings; however, previous research has indicated mixed results using visual cues for role designation.

**Methods:**

The purpose of this study was to explore the use of the red surgical hat as a visual cue of leadership during obstetric emergency simulation training. We used a mixed-methods design to analyze simulation-based education video and debriefing transcripts.

**Results:**

There was a statistically significant difference in the proportion of participants who declared leadership vs. those who donned the red hat. Participants were more likely to visually declare leadership utilizing a red surgical bouffant hat than to verbally declare leadership. Most participants indicated that observing the red hat to detect leadership in the operating room was more effective than when leaders used a verbal declaration to inform others who was leading.

**Conclusions:**

Our findings suggest that utilizing a visual cue of leadership with the red surgical bouffant hat improves participant perceptions of communication of the surgical team during an obstetrical critical event in a simulation environment.

## Introduction

Adapting to a critical event in labor and delivery requires the obstetrics team to immediately pivot from routine tasks to unexpected, life-saving tasks. In order for a team to respond to the rapidly evolving event, they first need to know that the situation is no longer routine, and team members need to establish a shared mental model about who is going to lead.

Previous research emphasizes that leadership is essential to a team’s ability to respond to a critical event [4, 11, 18]. In addition to nontechnical skills, such as delegating tasks, keeping “hands-off,” and maintaining situational awareness, the leader must be easily identifiable to all team members [1, 11]. Andersen et al. indicated that implicit/nonverbal transfer of leadership made it difficult for team members to clearly identify the team leader. However, more recent research focusing directly on leader identification suggests that team members prefer visual designation of leadership over verbal declaration of leadership [2]. Explicit Anesthesia and Nurse Distributed (EXPAND) Leadership is a modification of traditional CRM approaches [2]. EXPAND Leadership uses visual and verbal designation of leadership to explicitly signal that leadership has temporarily transferred to the circulating nurse and anesthesiologist, allowing the surgeon to focus on life-saving tasks such as controlling a postpartum hemorrhage. EXPAND Leadership is perceived to enhance communication between the surgeon, anesthesiologist, and circulating nurse. It also appears to improve team members’ ability to identify the leaders and reduce chaos in the operating room.

While the teamwork literature indicates a clearly designated leader is necessary during critical events [6, 8, 9], there is a paucity of research on methods to clearly identify a leader during an emergency. In most cases, guidance regarding the leadership designation suggests that the leader will verbally declare leadership during a critical event [3, 5].

While visual designation of leadership during a critical event in the operating room is still not well defined, there are examples of using visual cues to designate leadership in other settings. For example, Porter et al. [11] evaluated perceptions and experiences of teams’ nontechnical skills. Researchers reported that leaders attempted to use sticky labels, leadership hats, or vests to designate leadership. Although investigators noted challenges associated with visually designating leadership, they did not report on why some leaders opted to use a verbal declaration rather than a visual designation.

Although not specifically addressing leadership during a critical event, there are other instances where visual cues have been used to identify team roles in the operating room. Dr. Rob Hackett is credited with the #TheatreCapChallenge when he encouraged operating room staff to write their first name on their caps so team members could be identified by name or by their role [10]. Since then, several studies have investigated various methods to identify team members by their roles. Salaten et al. [14] measured the impact that using jackets to identify individuals’ roles had on teamwork during simulation training. Investigators found that using the jackets was associated with improved teamwork. However, a shortcoming of this study is that investigators excluded more than half of the videos from analysis because some of the trauma team did not wear identification jackets.

Other studies using visual cues to identify roles have reported mixed results. Rosen et al. [13] found that using different-colored head coverings helped operating room staff distinguish students from faculty. However, Sarcevic et al. [15] found that using stickers to distinguish roles during resuscitation was not successful because even when the role tags were used, the stickers were placed in different locations and orientations, making them difficult to see or read.

As part of the EXPAND Leadership training, educators believed that it was essential for all staff members to know who was leading during the critical event, including staff arriving to the operating room after the verbal declaration. Therefore, educators instructed the leaders to use visual designation and verbal declaration during simulated critical events. In addition to declaring “I will be the nurse lead” or “I will be the anesthesia lead,” the leaders would don a red hat. The red bouffant hat was chosen as the visual designation of leadership when pilot groups revealed verbal leadership designation was not reliable, and we asked groups for suggestions. The red hat was proposed and trialed, and it was clearly visible, readily available, easy to don, and easier to store in the anesthesia cart compared to a leadership vest or smock. Once we tried it a few times, it was clearly superior to verbal designation, and teams seemed comfortable with the concept, so it became the norm. The EXPAND training included a verbal declaration in addition to visual as this has been the traditional method of declaring leadership by rapid response teams. The purpose of this study was to investigate using a visual and verbal cue to designate the transfer of leadership during a simulated obstetrical emergency. Specifically, we wanted to gain insight into participants’ responses to using a visual and verbal cue to designate leadership. Additionally, we sought to determine if there were differences in individuals verbally declaring leadership compared to visually designating leadership by donning a red hat.

## Materials and methods

In this study, simulation serves as the investigational method for research. This study used data from a larger quality improvement project to explore labor and delivery operating room teams’ responses to using explicit distributed leadership. Full study methodology has been previously published [2]. Briefly, we used interpretive description [17] to understand participants’ perceptions of using visual and auditory cues to signal that an anesthesiologist and nurse will co-lead during a critical event in the obstetric operating room. Critical realism [7] underpinned our data analysis. Critical realism recognizes that aspects of reality, such as organizational hierarchies, exist outside of individuals, and that these entities can influence behavior whether individuals are aware of them or not. The research was conducted by a multidisciplinary team of researchers, which included individuals who were and were not involved in the development of the simulation.

The University of Kansas Health System is a large, urban tertiary care center with a well-established simulation center. The labor and delivery unit performed 2395 deliveries in 2021. The unit contains 11 beds, 7 triage or preoperative bays, and 2 operating rooms. Participants included all obstetrics and gynecology staff in the labor and delivery unit, including nurses, scrub techs, residents, fellows, and attending physicians (*N* = 160). The 3-h course included an orientation, simulation warm-up, didactic content, two simulation cases, and debriefings. Case 1 involved a pregnant woman arriving at L&D triage with a fetal heart rate in the 60 s. Upon being transported to the operating room, the woman becomes unresponsive. Case 2 involved a post-cesarean hysterectomy hemorrhage. In each of the cases, as soon as a team member recognizes a critical event is underway, the team member should declare, “This is a critical event.” Upon hearing that a critical event has been declared, the anesthesia provider and nurse use auditory and visual cues to signal a transition in leadership following our EXPAND model. The auditory cue includes the anesthesia provider’s declaration, “I will be the anesthesia co-lead,” and the circulating nurse’s declaration, “I will be the nurse co-lead.” Each leader should also don a red bouffant hat as a visual cue.

### Data collection

Our dataset originated from 58 recordings made between January 2019 and January 2020 of the simulations and post-event debriefing training sessions. Before participating in the course, all learners consented to recordings for educational and research purposes. The Institutional Review Board determined this study was not human subjects research, so IRB oversight was not required. Audio recordings from the debriefing sessions were professionally transcribed. Two members of the research team reviewed the transcripts for accuracy and replaced any names with pseudonyms Appendix (Table 4).

#### Instrumentation and rating

We developed an online instrument to assess the verbal declaration of leadership and visual designation of leadership. The primary rater (M. Z.) was a student participating in the KU Medical Center Education Experience (KEE). The secondary raters (H. W. and T. Z.) were graduate research assistants in the simulation laboratory. All raters were trained for standardization. The primary rater observed all videos (cases one and two) and used the online instrument to indicate if she detected a verbal and/or visual designation of leadership. If a designation of leadership was detected, the rater noted the timecode of the designation in the online instrument. Each secondary rater reviewed the videos for the cases using the online instrument results. Initial rater reliability was 92.2%.

### Quantitative analysis

Descriptive statistics were used to study the characteristics of the data. We used IBM SPSS Statistics for Windows, version 27 for all calculations (IBM Corp., 2020). The unit of analysis was at the individual level (whether an individual declared leadership). We conducted two-tailed *Z*-tests to determine if there were statistically significant differences in the proportions of participants who verbally declared leadership vs. donned the red hat, verbally declared leadership vs. received assistance donning the red hat, and donned the red hat vs. received assistance donning the red hat in cases 1 and 2.

We conducted two-tailed Fisher’s exact tests to determine if there were statistically significant differences between the proportions of participants who verbally declared leadership, donned the red hat, and received assistance donning the red hat in case 1 vs. case 2. The significance level was 0.05 for all statistical tests.

#### Debriefing data analysis

Four members of the research team (A. W., J. B., M. W., and S. P.) used a deductive qualitative framework approach to segment qualitative data. We used RQDA [12] to extract excerpts from debriefing transcripts that referred to declaring or designating leadership. A four-category coding framework included the following codes: seeing the visual designation of leadership, not seeing the visual designation of leadership, hearing the verbal declaration of leadership, and not hearing the verbal declaration of leadership. Subcategories that served to index the results were created. Frequency counts were used to describe the results. It should be noted that the verbal and visual designation of leadership was a standard debriefing topic in both simulation debriefings. Techniques to enhance the trustworthiness and credibility of data analysis [16] included immersion and prolonged engagement with the data. Additionally, peer debriefing was used throughout the analysis process. Finally, we used mixed methods to enhance the trustworthiness of this research. By combining qualitative and quantitative analysis, we could balance the insight obtained by using participants’ words with the objectivity of reviewing video tape to observe participants’ behaviors. Differences in coding were resolved by consensus.

## Results

One-hundred sixty participants attended 15 3-h training sessions between 2019 and 2020. Demographic information is provided in Table 1. We analyzed 30 simulations and 28 debriefing sessions. Data from two debriefing sessions could not be analyzed due to poor recording quality.

**Table 1:** Participant demographic information

Profession	*n* (% of N)	Number of learners who participated in more than one training
Anesthesia attendings	15 (9%)	0
Anesthesia residents	9 (6%)	2
CRNAs	11 (7%)	4
OB-Gyn attendings	24 (15%)	2
OB-Gyn fellows	2 (1%)	0
OB-Gyn residents	17 (11%)	0
RNs	71 (44%)	1
Scrub technicians	11 (7%)	4
	***N* = 160 (100%)**	**14**

### Simulation observations

#### Case 1

Six (20.0%) participants out of a possible 30 leadership opportunities verbally declared leadership during the first case. Twenty-five (83.3%) out of a possible 30 opportunities visually designated leadership during the first case using the red hat. Individuals assisted other participants with the red hat 13 (43.3%) times. Most participants did not receive assistance donning the red hat. Results from the statistical analyses are provided in Table 2. There was a statistically significant difference in the proportion of leaders who verbally declared leadership compared to donning the red hat. There was also a statistically significant difference in the proportion of leaders who donned the red hat compared to those who received assistance donning the red hat.

**Table 2:** Group comparisons

First case in comparison	Second case in comparison	*p*-value
Case 1: Leader declared “I will lead”	Case 1: Leader donned red hat	< .001*
Case 1: Leader declared “I will lead”	Case 1: Leader received assistance donning hat	.065
Case 1: Leader donned red	Case 1: Leader received assistance donning hat	< .001*
Case 2: Leader declared “I will lead”	Case 2: Leader donned red hat	< .001*
Case 2: Leader declared “I will lead”	Case 2: Leader received assistance donning red hat	0.774
Case 2: Leader donned red hat	Case 2: Leader received assistance donning red hat	< .001*
Case 1: Leader declared “I will lead”	Case 2: Leader declared “I will lead”	0.603
Case 1: Leader donned red hat	Case 2: Leader donned red hat	1.00
Case 1: Leader received assistance donning red hat	Case 2: Leader received assistance donning red hat	0.628

Two-tailed *Z*-tests using the exact binomial method were used to compare proportions within cases. Two-tailed Fisher’s exact tests were used to compare proportions between case 1 and case 2. *Significant at .05 threshold

#### Case 2

Seven (23.3%) participants verbally declared leadership compared to 29 (96.7%) participants who visually designated leadership during the second case. Individuals assisted other participants with the visual declaration of leadership five (16.7%) times. Analysis revealed that there was a statistically significant difference in the proportion of leaders who verbally declared leadership compared to donning the red hat. There was also a statistically significant difference in the proportion of leaders who donned the red hat compared to those who received assistance donning the hat. There were no statistically significant differences when we compared verbal declaration, donning the red hat, and receiving assistance donning the red between cases 1 and 2.

### Debriefing (qualitative) results

We identified 77 excerpts from 28 debriefing sessions in which participants referenced “seeing the visual designation of leadership,” “not seeing the visual designation of leadership,” “hearing the verbal declaration of leadership,” and “not hearing the declaration of leadership.” Table 3 provides a summary of the frequencies.

**Table 3:** Summary of debriefing results

	Categories			
	Seeing^a^	Not seeing^b^	Hearing^c^	Not hearing^d^
**Total** *N* **= 83 (100%)**	57 (*n* = 69%)	8 (*n* = 10%)	7 (*n* = 8%)	11 (*n* = 13%)
**Simulations**^**e**^ *N* **= 28**	19 (*n* = 68%)	4 (*n* = 14%)	6 (*n* = 21%)	10 (*n* = 36%)
**Excerpts**	Participant 1: I like the red hats…It just helps with a lot of the communication Participant 2: I want to second that. I like knowing kind of who to go to, who to look for, for what's my direction?	I was looking for a hat, I didn't see one	Faculty: I have to apologize. I did not hear declaration of, I'm anesthesia lead and I'm nurse lead Learner: I did	I assume that you took over as anesthesia lead Um, but um, I don't really ever hear you saying I'm the anesthesia lead
	I really liked the visual designation of the leaders in the room and I think that's really helpful	I was doing compressions for a while, so I feel like I never looked up. So I had no idea who was the lead	Faculty: Did anybody actually hear him declare the leadership? Learner: I did, but I was standing right next to him	Faculty: Did anybody hear him declare himself as the anesthesia lead? Participant 1: No Participant 2: I just know he was because he had on a red hat

^a^Seeing and then visual designation of leadership

^b^Not seeing the visual designation of leadership

^c^Hearing the verbal declaration of leadership

^d^Not hearing the verbal declaration of leadership

^e^Frequency of cases associated with each domain

We identified 53 (68%) excerpts from 19 debriefing sessions that referred to “seeing the visual designation of leadership.” Forty-two (79.2%) of the excerpts indicated that seeing the red hat helped the participant in some way. For example, several participants described how the visual designation of leadership helped with taskwork, “I like being able to have the red hat to go to, to find out exactly what I need to do.” Another participant associated using the red hat with improved communication, “I think part of this is forcing us to have better communication on each side of the curtain. I think the red hat is the cue of who is the person that we need to be talking to.” While most comments about the red hats described ways in which the red hats contributed to teamwork, 11 (20.8%) of the excerpts were simply confirmations that a participant saw the visual designation of leadership.

We identified eight excerpts from five debriefing sessions associated with “not seeing the visual designation of leadership.” Four (50%) of the excerpts from this category suggest that the participant did not notice the red hat. For example, when a faculty member asked, “How did the red hats work on the surgery side?” one participant responded, “I didn't really notice got to be honest.” Two excerpts (25%) from this category indicated that some participants did not see the red hats because they were either busy trying to identify the cause for the critical event or were involved in resuscitating the patient. “(I’m) looking at a uterus, the pathology that led her to not being responsive, and in trying to identify that, I didn't look up.” Another participant stated that she was “doing compressions for a while, so I feel like I never looked up. So, I had no idea who was the lead.” One excerpt (12.5%) from this category indicated that the participant did not remember if she saw the red hat or not. Another excerpt from this category (12.5%) indicated that the participant looked for the red hat when she entered the room, but she “didn’t see one.”

We identified seven excerpts from five debriefing sessions associated with “hearing the declaration of leadership.” Two (28.6%) excerpts from this category indicated that a participant was able to hear the declaration of leadership, “Maybe it's a little harder to hear if you're…facing the back of the room.” But I heard Dr. Smith say, “I'm Dr. Smith, the anesthesia lead.” Two participants (28.6) indicated they heard the declaration, but they did not know who the leader was, “because we're surgeons, we're looking into the belly. You have a mask on, I don't know who's saying it, and I definitely don't recognize the voice.” One excerpt (14.3%) indicated that the participant heard the declaration because of their proximity to the leader, “I did [hear], but I was standing right next to him.”

We identified nine excerpts from nine debriefing sessions associated with “not hearing the declaration of leadership.” Six (66.7%) excerpts from this category merely indicated that participants could not hear the declaration. Two (22.2%) excerpts described not hearing the declaration because the participant was focused on something else, “At one point I was like ‘Who's the lead?’ And they are like, ‘Oh we already called the lead.’ But I…missed that because we were getting her ready to do the surgery.” One (11.1%) excerpt referred to not hearing the verbal declaration but knowing who the leader was because the participant saw the red hat.

## Discussion

### Principal findings

Our study found that participants in an obstetric critical event simulation responded differently to using a visual designation of leadership compared to using a verbal declaration of leadership. The participants in our sample were more likely to declare leadership using the visual cue than they were to verbally declare leadership. Not surprisingly, participants were more likely to indicate that they had seen the visual declaration of leadership than they were to report that they had heard the verbal declaration during an obstetrical emergency simulation.

### Results in the context of what is known

This study makes a significant contribution to the literature by focusing on an area of obstetrical crisis resource management training that has received little attention. While crisis resource management training is well received, our approach is novel in that during an obstetric critical event, a transfer of leadership occurs. The anesthesiologist and circulating nurse declare leadership using visual and verbal cues, with the success of the visual cue as a key component of EXPAND leadership. Our findings contrast with previous research indicating leaders were more likely to verbally declare leadership than visually declare leadership [11]. In our study, the proportion of leaders who visually designated leadership was significantly greater than that of leaders who verbally declared leadership. Additionally, during debriefings, learners were more likely to report seeing the red hat than they were to discuss that they heard the verbal declaration of leadership. With respect to learners’ comments during the debriefings, it should be noted that some learners may have heard the verbal designation of leadership but did not mention it during the discussion. Additionally, more comments may have focused on the visual cue than the verbal cue because the visual designation was a novel component of the training. However, the results from the analysis of the debriefing discussion are consistent with the quantitative analysis of the observations of the simulation videos.

Our findings that leaders were more likely to use a visual cue to declare leadership than verbally declare leadership may have differed from Porter et al.’s findings for several reasons. Our study specifically focused on the designation of leadership during a simulated obstetrical emergency. Learners were observed immediately after receiving training which instructed leaders to verbally and visually designate leadership. Porter et al. conducted a qualitative study evaluating perceptions and experiences of emergency personnel’ experiences using the Team Emergency Assessment Measure to rate nontechnical skills in clinical resuscitation events from two different emergency departments, and it is not clear if staff were trained to use a visual cue to designate leadership. However, investigators did point out that the physical designation of leadership was “an area requiring attention despite various efforts to allocate and label the leader.” Our study and Porter et al.’s study focused on emergency events that were “changeable.” Additionally, our findings may have differed from Porter et al. because they investigated reflections following actual resuscitation events, and our study involved observations made during a simulated event and debriefing discussions.

The findings of this study build on the conclusions of Rosen et al. [13], who found that using different colored head coverings improved the ability of team members to discern roles in the operating room. Using hats to designate roles during a critical event appears to be more effective than using stickers, as previous research indicates that stickers were not consistently visible [15]. In real-life settings, it will be essential for team members to know where the red hat is stored, as previous studies have reported problems associated with locating role tags during an emergency [15].

Our findings suggest that the use of a red surgical hat as a visual cue to designate leadership improves teams’ ability to recognize who is leading during a critical event. In a simulated setting, the visual declaration improved teamwork and communication during emergencies [2]. The obstetrician-gynecologists who participated appreciated the ability to focus on the surgical field and have designated contacts to direct their communication. Broski et al. [2] found that in the hemorrhage simulation, several surgeons indicated that using the red hat to designate leadership helped them know who to communicate with, “Because we're looking into surgeons, we're the belly. You have a mask on, I don't know who's saying [who will lead], and I definitely don't recognize the voice… and I'm not going to recognize the voice because I have to be paying attention… So, having a red [hat]… To look up, I don't necessarily have to hear [the declaration of leadership]; I just need to know who it is.”

Our study has several strengths. The findings of this study address an important gap in the literature since there is scant research on using a visual cue to identify leadership during a simulated obstetrical emergency. Utilizing a striking visual cue of leadership in an environment filled with uniform appearances elevates a simple red bouffant to an obvious coordinated leadership designation. One strength of our study is the multidisciplinary approach to the development of the intervention which resulted in strong interdisciplinary buy-in. Our research extends the literature of previous studies on role tagging with a new visual focus. Another strength of our study is utilizing mixed methods by supplementing the qualitative data with objective outcomes.

Our study has several limitations. This study was carried out in a single academic institution; therefore, the results may not generalize to other institutions. To ensure representation of all services in every simulation, some learners attended training more than once, and it is possible that individuals participating multiple times could influence the findings. Our study took place in a simulated environment. We do not have data for actual obstetric emergencies, and we do not know if these findings would be replicated in other settings. Future research should investigate emergencies across multiple locations and settings, including how this integrates on labor and delivery outside of the simulated environment. It can also explore why it was easier to don the red hat as a visual cue over making a verbal declaration of leadership.

## Conclusion

Despite the importance of team members identifying the leader during an emergency in the operating room, few studies have explored ways in which a visual cue to designate leadership can enhance communication. Our findings suggest the chaos of an obstetric emergency benefits from a striking visual cue in the red surgical bouffant hat to communicate leadership.

## Data Availability

The quantitative data analyzed during the current study are available from the corresponding author on reasonable request. The qualitative data analyzed during the current study are not publicly available due to the need to protect the privacy of study participants. The processed data is available from the authors upon reasonable request.

## Appendix

**Table 4:** Data collection tool

Observation guidance	Data collection
Date	
Case	
Leader 1 declared leadership — minutes	
Leader 1 declared leadership — seconds	
Leader 1 donned red hat — minutes	
Leader 1 donned red hat — seconds	
Leader 1 received assistance donning hat — minutes	
Leader 1 received assistance donning hat — seconds	
Leader 1 verbally declared leadership — 1 = yes, 2 = no, 3 = unsure	
Leader 1 donned hat	
Leader 1 received assistance donning hat	
Leader 2 declared leadership — minutes	
Leader 2 declared leadership — seconds	
Leader 2 donned hat — minutes	
Leader 2 donned hat — seconds	
Leader 2 received assistance donning hat — minutes	
Leader 2 received assistance donning hat — seconds	
Leader 1 verbally declared leadership 1 = yes, 2 = no, 3 = unsure	
Leader 1 donned the red hat 1 = yes, 2 = no, 3 = unsure	
Leader 1 received assistance donning the red hat 1 = yes, 2 = no, 3 = unsure	
Leader 2 verbally declared leadership 1 = yes, 2 = no, 3 = unsure	
Leader 2 donned the red hat 1 = yes, 2 = no, 3 = unsure	
Leader 2 received assistance donning the red hat 1 = yes, 2 = no, 3 = unsure	

Leader 1 was the first individual observed to declaring leadership or don the hat. Leader 2 was the second individual observed to declare leadership or don the hat. In analysis, any instance in which the observer was unsure about an observation was counted as “no.” For presentation purposes, the data collection sheet is displayed in portrait orientation. During data collection, the orientation of the sheet was horizontal

## References

[1] Andersen PO, Jensen MK, Lippert A, Østergaard D (2010). Identifying non-technical skills and barriers for improvement of teamwork in cardiac arrest teams. Resuscitation.

[2] Broski J, Tarver S, Krase K, Petersen S, Wolverton A, Winchester M, Berbel G, Zabel T, Warren H, Lineberry M. Integrating simulation and interpretive description to explore operating room leadership: critical event continuing education. Adv Health Sci Educ. 2023;28(4):1–34. 10.1007/s10459-023-10212-3 37022534

[3] Butscher. Leadership and crisis management. In: Murugan R, Darby JM, editors. Rapid response system: a practical guide. New York: Oxford University Press; 2018.

[4] Castelao FE, Russo SG, Riethmüller M, Boos M (2013). Effects of team coordination during cardiopulmonary resuscitation: a systematic review of the literature. J Crit Care.

[5] Colibri Healthcare, LLC (2023). Pennsylvania Nursing Continuing Education.

[6] Cracknell A, Cooper N. Communication in clinical teams. In: Cooper N, Frain J, editors. ABC of clinical communication. Hoboken: Wiley; 2018.

[7] Ellaway RH, Kehoe A, Illing J (2020). Critical realism and realist inquiry in medical education. Acad Med.

[8] Guise JM, Segel S (2008). Teamwork in obstetric critical care. Best Pract Res Clin Obstet Gynaecol.

[9] Martins J, Sousa A, Abrantes A, Pinto C, Gomes C, Martins D, Coutinho V, Baptista R, Oliveira L, Fernandes M (2017). Communication and leadership in emergency situations: systematic literature review and recommendations for practice. Clin Nurs Stud.

[10] Nelson A. #TheatreCapChallenge: Safety changes and the PatientSafe Network. O&G Magazine; 2019. https://www.ogmagazine.org.au/21/3-21/theatrecapchallenge-safety-changes-and-the-patientsafe-network/.

[11] Porter JE, Cant RP, Cooper SJ (2018). Rating teams’ non-technical skills in the emergency department: a qualitative study of nurses’ experience. Int Emerg Nurs.

[12] Ronggui H. RQDA: R-based qualitative data analysis [Computer software]. 2012. http://rqda.r-forge.r-project.org/.

[13] Rosen DA, Criser AL, Petrone AB, Jackson E, Bowers J (2019). Utilization of a role-based head covering system to decrease misidentification in the operating room. J Patient Saf.

[14] Saleten M, Laitselart P, Martinez T, Descamps C, Debien B, Boutonnet M, Pasquier P (2022). Who’s Who in the trauma bay? Association between Wearing of identification jackets and trauma teamwork performance: a simulation study. J Emerg Trauma Shock.

[15] Sarcevic A, Marsic I, Waterhouse L, Stockwell D, Burd R (2011). Leadership structures in emergency care settings: a study of two trauma centers. Int J Med Informatics.

[16] Stahl NA, King JR (2020). Expanding approaches for research: understanding and using trustworthiness in qualitative research. J Dev Educ.

[17] Thorne S (2016). Interpretive description: qualitative research for applied practice.

[18] Yeung JH, Ong GJ, Davies RP, Fang G, Perkins GD (2012). Factors affecting team leadership skills and their relationship with quality of cardiopulmonary resuscitation*. Crit Care Med.

